# FS-YOLO: A Lightweight Insulator Defect Detection Method Based on Multi-Level Feature Fusion and Localization Quality Estimation

**DOI:** 10.3390/s26185876

**Published:** 2026-09-16

**Authors:** Congjie Wen, Zhiliang Zhu, Yijian Weng, Zekai Cai, Jiacheng Wang

**Affiliations:** 1 College of Electrical and Electronic Engineering, Wenzhou University, Wenzhou 325000, China; 24451249029@stu.wzu.edu.cn (C.W.); 24461251009@stu.wzu.edu.cn (Y.W.); 25451251001@stu.wzu.edu.cn (Z.C.); 25451250029@stu.wzu.edu.cn (J.W.); 2 Wenzhou Key Laboratory of Unmanned Intelligent Systems and Equipment, Wenzhou University, Wenzhou 325000, China

**Keywords:** insulator defect detection, YOLO11n, lightweight, multi-level feature fusion, localization quality estimation

## Abstract

To address the challenges of low localization accuracy for insulator defects in complex inspection scenarios and excessive model redundancy that hinders edge deployment, this paper proposes FS-YOLO, a lightweight defect detection algorithm. First, a C3k2 ConvFormer Gated Linear Unit (C3k2-CFGLU) module replaces the original backbone, using depthwise separable convolution and gated linear units to enhance defect feature representation while reducing computation. Second, a Multi-Branch Multi-Scale Feature Pyramid Network (MBMSFPN) neck improves defect target perception via multi branch auxiliary connections that strengthen high-level and low-level feature interaction. Third, a Shared Convolution Detection Head (SCDH) reduces parameters through multi-scale shared convolutions and group normalization, and incorporates a localization quality estimation mechanism to offset lightweight induced accuracy loss. Finally, layer adaptive sparsity for magnitude-based pruning (LAMP) removes redundant channels, compressing model size and improving efficiency. Experiments show that FS-YOLO achieves 93.0% precision, 88.3% recall, 92.9% mAP@0.5, and 62.9% mAP@0.5:0.95, with only 0.88M parameters and 4.1 GFLOPs. Compared with the baseline, parameters and computation drop by 65.9% and 34.9%, while mAP@0.5 increases by 5.7%. Against other mainstream YOLO variants, FS-YOLO offers superior accuracy efficiency trade offs, offering a promising reference for the intelligent development of power line inspection.

## 1. Introduction

The power grid is undergoing rapid, healthy, and sustainable development worldwide. As national critical infrastructure, its safe and stable operation is essential to economic and social development. Insulators, as indispensable components of transmission lines, are exposed to complex and changing natural environments for long periods and must withstand combined electrical, thermal, mechanical, and environmental stresses. Their integrity directly affects the reliability of the entire power grid. Once defects such as breakage or pollution-flashover occur, they can readily lead to partial discharge, current leakage, or even breakdown accidents, resulting in considerable economic losses and severe social consequences [1,2].

Traditionally, insulator inspection has relied primarily on manual visual inspection or telescope observation. This approach is not only inefficient and labor-intensive but also highly dependent on the subjective experience of inspectors and external environmental conditions, which leads to high rates of missed detections and false alarms. In recent years, with the maturity of unmanned aerial vehicle (UAV) technology and digital photography, UAV inspection combined with high-definition cameras has become the mainstream mode of power line inspection. This mode produces massive amounts of on-site image data, making manual screening impractical. Consequently, there is an urgent need for intelligent automatic detection technologies that can quickly and accurately identify defective insulators.

Methods for insulator defect detection can be broadly divided into traditional image processing approaches and deep learning-based methods. Traditional image processing attempts to construct a universal rule that separates insulator defects from the background. For example, Tiantian Y et al. [3] proposed a method that fuses histogram of oriented gradient (HOG) and local binary pattern (LBP) features after reducing their dimensionality with principal component analysis (PCA). Yongjie Z et al. [4] identified the target region of insulators on the basis of spatial features and established detection rules according to the spatial feature differences between faulty and non-faulty insulators. Zhai Y et al. [5], considering the special structure of insulators, used morphology as an entry point to jointly exploit the capabilities of saliency and morphology. Wu Q et al. [6] proposed a texture segmentation algorithm that extracts insulator texture features with the gray-level co-occurrence matrix (GLCM) and accelerates computation with the fast gray-level co-occurrence integrated algorithm (GLCIA) to achieve efficient contour evolution. However, the stability of these techniques is limited, and they cannot meet the precise detection requirements of insulator defects in power line inspection.

Deep learning-based object detection algorithms can be broadly divided into two categories. The first is the two-stage approach, represented by Fast R-CNN [7] and R-FCN [8]. The second is the one-stage approach, which predicts the types and positions of objects with a single forward pass of a convolutional neural network (CNN), including SSD [9], YOLO, FSSD [10], and other variants. Prior research has largely focused on improving and applying general detection models. For example, MobileNetV3 [11] employs automated neural architecture search (NAS), combining platform-aware NAS with the NetAdapt algorithm to enhance performance and efficiency. The RevCol model [12] consists of multiple copies of sub-networks, termed columns, connected by multi-level reversible connections, which endow it with behavior distinct from traditional networks. Lu Y et al. [13] adopted GhostNet combined with a newly designed lightweight channel-spatial attention (LCSA) mechanism as the backbone to strengthen feature extraction, and introduced GSConv and C3Ghost modules to reduce the parameter and computational burden. Li D et al. [14] designed LiteYOLO-ID, a lightweight detection model based on YOLOv5s, which optimizes feature extraction and achieves model compression; after TensorRT optimization, it runs at 20.2 frames per second on the Jetson TX2 NX. Liu Y et al. [15] proposed LDIDD, an insulator defect detection method based on YOLOv8, which incorporates depthwise separable convolutions into the YOLOv8 framework to alleviate the deployment difficulty caused by model complexity on UAV devices. Chen Q et al. [16] replaced the original C2PSA module with a CSPCA module incorporating coordinate attention to enhance spatial perception, and introduced a composite loss combining Wise-IoUv3 and focal loss to accelerate convergence and improve accuracy. Gujing H et al. [17] embedded the ECA-Net channel attention mechanism into the feature extraction layers and the PANet path aggregation network to improve small-target recognition while making the model more lightweight. Wang Q et al. [18] proposed YOLOLS for scenarios with limited computational resources and stringent latency constraints, integrating GhostConv and restructuring the C2f module into a ResNet-RepConv architecture to reduce complexity and improve inference speed. Wang J et al. [19] designed TRS-YOLO, introducing a receptive field attention convolution module (RFAConv), a spatial and channel synergistic attention module (SCSA), and a mobile inverted bottleneck block (MBConv), providing a lightweight solution for edge deployment. Hu C et al. [20] proposed DVW-YOLO, which uses dynamic elliptical convolution to focus more efficiently on elliptical features and substantially reduces redundant feature extraction and multi-scale fusion. Li J et al. [21] proposed A2MADA-YOLO, which, through implicit adversarial domain adaptation, multi-scale optimization, and explicit attention alignment, considerably improved the mAP0.5 of YOLOv7, v8, and v9 on both known and unknown foggy target domains, thereby validating its effectiveness in learning domain-invariant features. Shen P et al. [22] proposed LDDFSF-YOLO11, which incorporates multi-module improvements and channel pruning, achieving a 7.1% mAP improvement over YOLO11n on a self-built insulator dataset with a model size of only 4.2 MB, enabling lightweight and efficient detection of small-sized defects under complex backgrounds. Despite these promising results, such methods often achieve limited accuracy on small objects such as insulator defects. Moreover, because insulators frequently appear in complex backgrounds, existing models remain highly susceptible to interference from redundant background information. At the same time, the large computational requirements of current models make it difficult to meet the demands of industrial edge deployment. Therefore, considerable challenges remain in compressing model size while simultaneously improving accuracy and interference resistance.

To address the above issues, this paper proposes a lightweight insulator defect detection model based on YOLO11n, named FS-YOLO. The main contributions are as follows:

- A C3k2-CFGLU module is designed as a feature extraction unit tailored for lightweight fine-grained object detection. By effectively integrating the spatial mixing capability of ConvFormer and the channel modulation capability of CGLU into the C3k2 framework, this module achieves accurate extraction and enhancement of defect features in complex backgrounds.
- A MBMSFPN module is designed as an advanced neck architecture that integrates multi-scale feature extraction, efficient feature alignment, and adaptive weighted fusion. This design constructs denser and more flexible information interaction paths and introduces learnable weights to optimize the fusion process, effectively addressing the shortcomings of traditional FPN in defect object detection and multi-scale feature balancing, thereby providing higher-quality feature pyramids for subsequent detection heads.
- A SCDH module is designed as a lightweight detection head structure using shared convolutions, which is further enhanced by a localization quality estimation mechanism to improve the credibility of predicted boxes and compensate for the localization accuracy loss caused by lightweight design. These two components complement each other, providing an efficient and accurate detection head design solution for the insulator defect detection task.
- To address the hardware resource constraints of edge devices in industrial deployment, the layer-adaptive sparsity for the magnitude-based pruning (LAMP) technique is employed to remove redundant channels in the model, further optimizing the model structure and improving model performance.

## 2. Related Works

### 2.1. YOLO11 Algorithm

**Figure 1:**
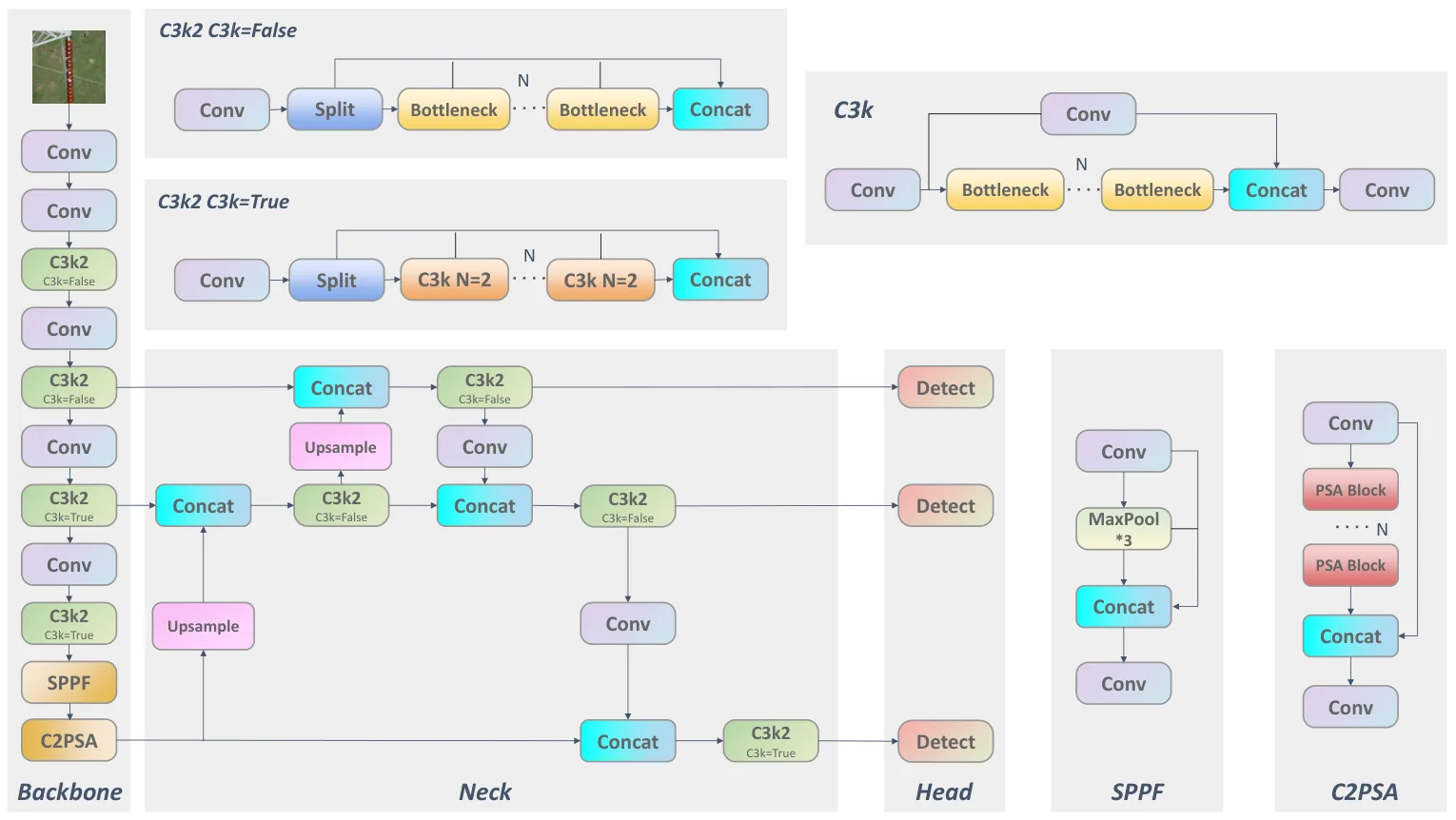
The basic architecture of YOLO11.

YOLO11 [23] is an object detection model released by Ultralytics. It continues the core concept of single-stage detection of the YOLO series, achieving a further balance between detection accuracy and model compression while maintaining real-time inference capability. Its architecture is shown in Figure 1. The model incorporates systematic innovations in architectural design. The backbone network adopts an optimized CSPDarknet structure, introducing the C3k2 module and the C2PSA attention module, effectively enhancing feature extraction capability. The neck network employs an improved feature pyramid network structure, achieving efficient multi-scale feature interaction through weighted feature fusion and cross-stage connections. The detection head adopts a decoupled design, separating the classification and regression tasks. With its lightweight characteristics and efficient inference speed, YOLO11 provides an ideal baseline framework for constructing higher-precision lightweight detection systems.

### 2.2. Weighted Bidirectional Feature Pyramid Network

In object detection, effectively fusing multi-scale features extracted by the backbone network is crucial for handling objects of varying sizes. The traditional Feature Pyramid Network (FPN) [24] introduces a top-down path for feature fusion, but its unidirectional information flow limits the fusion effectiveness. To address this issue, PANet [25] adds an additional bottom-up path based on FPN, improving feature propagation capability but also considerably increasing the parameter count and computational overhead.

**Figure 2:**
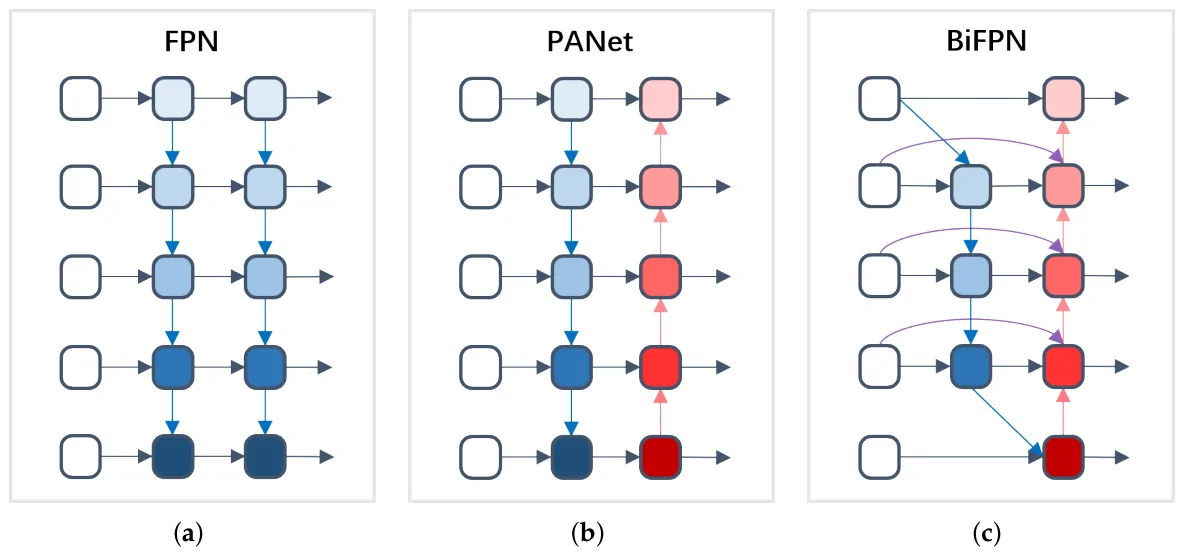
Architectures of the (**a**) FPN; (**b**) PANet; (**c**) BiFPN.

To solve the above problems, Tan et al. [26] proposed the Weighted Bidirectional Feature Pyramid Network (BiFPN). The design of BiFPN follows three main principles. First, the cross-scale connection topology is simplified by removing nodes with only a single input edge, as such nodes contribute little to feature fusion. Second, additional skip connections are added between the original input nodes and output nodes at the same level to fuse more features with almost no increase in computational cost. Third, each bidirectional (top-down and bottom-up) path is treated as one feature network layer, and this layer is repeatedly stacked multiple times to achieve higher-level multi-scale feature fusion. The network architectures of FPN, PANet, and BiFPN are shown in Figure 2.

In addition to structural improvements, BiFPN introduces learnable weights to fuse features of different resolutions. Instead of simply summing the input features, BiFPN assigns a trainable weight to each input, enabling the network to autonomously learn the importance of features at different scales. To avoid the high latency issue caused by Softmax normalization, BiFPN adopts a fast normalized fusion method:

(1) $$O=\sum_{i}\frac{w_{i}}{\epsilon +\sum_{j}w_{j}}\cdot I_{i}.$$

where $w_{i}\geq 0$ is a learnable weight, ϵ is a very small constant to prevent numerical instability, and $I_{i}$ represents the input features. This method constrains the normalized weights to a range between 0 and 1 without requiring Softmax operations, considerably improving inference speed while maintaining comparable accuracy.

By combining an efficient bidirectional connection structure with a weighted feature fusion mechanism, BiFPN outperforms traditional feature fusion methods such as FPN, PANet, and NAS-FPN in both accuracy and efficiency. As the core feature network of the EfficientDet series of models, BiFPN provides critical support for achieving scalable and high-performance object detection.

### 2.3. ConvFormer: A Pure Convolutional Model Based on MetaFormer

In the field of computer vision, the success of Transformers was long attributed to their self-attention mechanism. However, Yu et al. [27] proposed the MetaFormer, a general architecture, and pointed out that the key factor is the architecture itself rather than the specific Token Mixer. MetaFormer is an abstract framework in which each block consists of a normalization layer, a Token Mixer (for token-to-token information interaction), and a channel MLP (for feature transformation), connected via residual connections.

**Figure 3:**
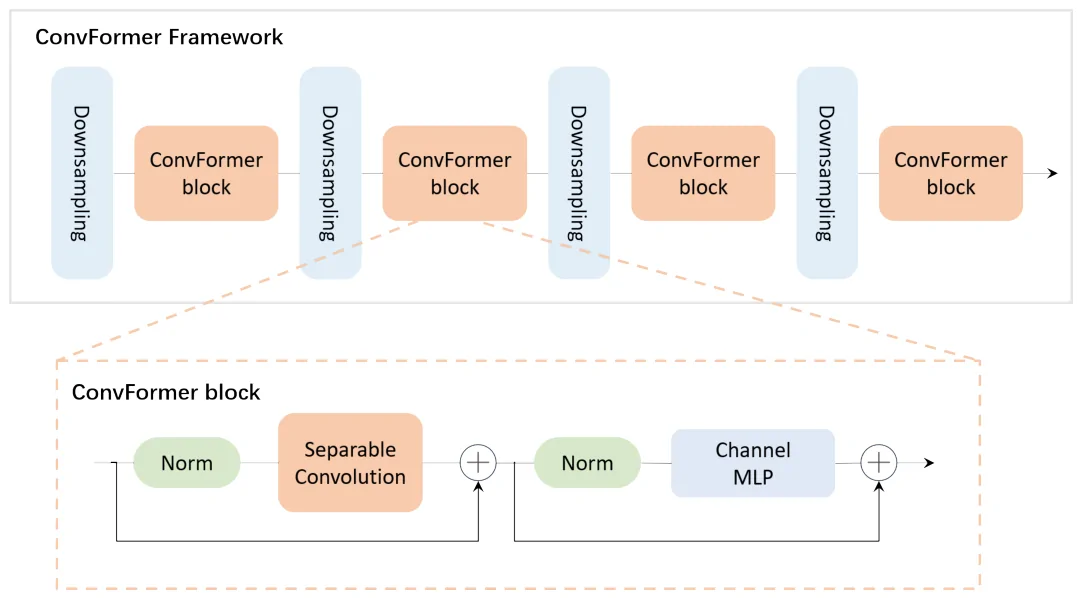
Architectures of the ConvFormer.

To validate the potential of MetaFormer, Yu et al. did not design novel Token Mixers but instead directly instantiated the architecture using classical operations that had existed for several years. Among these instantiations, ConvFormer is the model obtained by specifying depthwise separable convolution as the Token Mixer. The network architecture of ConvFormer is shown in Figure 3. ConvFormer follows the inverted residual structure proposed in MobileNetV2 [28]: it first expands the channel dimension through pointwise convolution, then performs depthwise convolution (typically with a 7 × 7 kernel), and finally compresses the channel dimension back to the original size through another pointwise convolution. The entire Token Mixer module employs non-linear activation functions and operates within the dual residual connection framework of MetaFormer. Without introducing any novel Token Mixer, ConvFormer constructs a competitive high-performance model relying solely on the MetaFormer architecture and classical convolutional operations. This further confirms the central role of the MetaFormer architecture itself in vision tasks and provides a simple yet powerful baseline for future network architecture design.

## 3. Proposed Method and Experimental Setup

**Figure 4:**
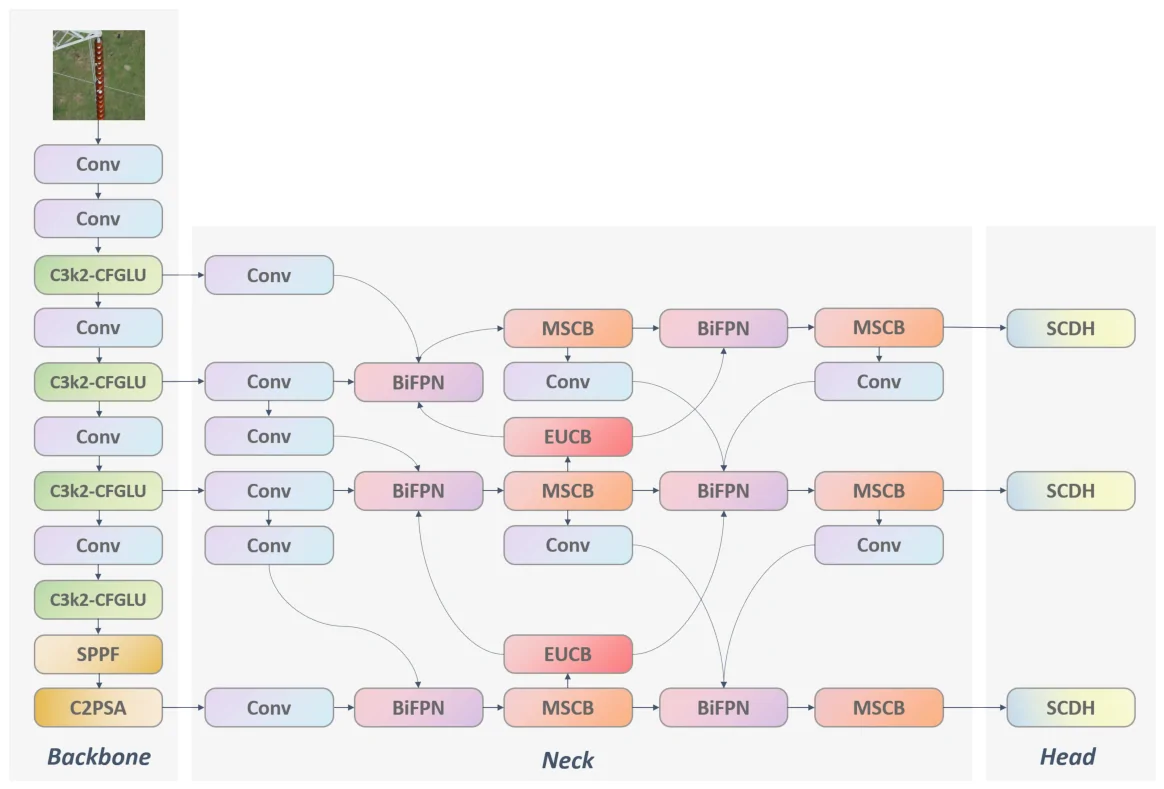
The overall architecture of the proposed FS-YOLO model.

Based on the YOLO11 model framework, this paper proposes a lightweight improved model for insulator defect detection, named FS-YOLO, by optimizing three core components—the backbone network, neck network, and detection head—along with the LAMP channel pruning technique. The architecture is shown in Figure 4. To address the characteristics of small defect scales and strong background interference in insulator defect detection, a C3k2-CFGLU module is designed in the backbone network. By employing depthwise separable convolution to simulate the spatial mixing capability of Transformers and combining it with the gated linear unit CGLU to achieve channel-wise dynamic modulation based on local neighborhood features, the module enhances fine-grained feature representation. To optimize multi-scale feature fusion, an MBMSFPN module is designed to replace the original neck structure. This module enhances the interaction between shallow spatial details and deep semantic information through multi-branch auxiliary connections, and improves sensitivity to small-scale defects by utilizing multi-scale depthwise convolution blocks and efficient upsampling. To reduce computational redundancy in the detection head, a lightweight detection head named SCDH is designed. It considerably reduces the parameter count through multi-scale shared convolutions and group normalization, and generates reliable localization quality scores using the statistical characteristics of bounding box distributions to compensate for accuracy loss. Finally, the layer-adaptive sparsity for the magnitude-based pruning (LAMP) technique is introduced to eliminate redundant channels in the model, greatly reducing the computational cost and parameter count.

It should be noted that there exists an inherent tradeoff among detection accuracy, parameter count, FLOPs, and inference latency. In general, increasing the model capacity (i.e., more parameters and higher computational cost) tends to improve detection accuracy, but simultaneously increases model size and latency, which is detrimental to edge deployment. Conversely, aggressively reducing model complexity may lead to a considerable degradation in detection performance, especially for small defect targets. Therefore, the design philosophy of FS-YOLO is to seek an optimal balance: on the one hand, the lightweight design at the module level reduces the parameter count and computational cost while enhancing feature representation capability; on the other hand, the LAMP pruning technique further compresses the model to meet the deployment budget, while the localization quality estimation mechanism compensates for the potential accuracy loss caused by lightweight design. Through this systematic optimization across multiple dimensions, a favorable balance between accuracy and efficiency is achieved. The following sections provide detailed descriptions of the aforementioned C3k2-CFGLU module, MBMSFPN module, SCDH lightweight detection head, and LAMP pruning technique.

### 3.1. Deployment-Oriented Design Constraints

To ensure practical deployability in real-world UAV-based power line inspection, the proposed FS-YOLO is explicitly designed to operate on resource-constrained edge devices. We select the NVIDIA Jetson Orin Nano 8GB as the representative deployment platform, which is widely adopted in industrial inspection systems. Based on its hardware specifications (GPU peak throughput of up to 20 TOPS INT8, limited memory bandwidth, and strict onboard storage limits) and the real-time processing requirements for continuous video streaming (at least 30 FPS), we define the following measurable hard constraints that must be satisfied by the final model:

(1) Model storage size ≤ 3.0 MB;
(2) Computational complexity ≤ 5.0 G FLOPs;
(3) Number of parameters ≤ 1.0 M;
(4) Inference latency ≤ 33.3 ms per image (equivalent to a throughput of at least 30 FPS).

### 3.2. C3k2-CFGLU Module

In the original YOLO11n, the backbone adopts an efficient C3k2 structure to pursue high speed. However, for tasks requiring fine-grained recognition of defect targets such as insulator defect detection, the limited receptive field and constrained feature representation capability of standard convolutions may become bottlenecks. The model is prone to missed detection of defect targets or false alarms in complex backgrounds. Therefore, the core motivation for designing the C3k2-CFGLU module proposed in this paper is to reduce computational burden while enhancing the model’s ability to extract fine-grained features and focus on critical regions.

**Figure 5:**
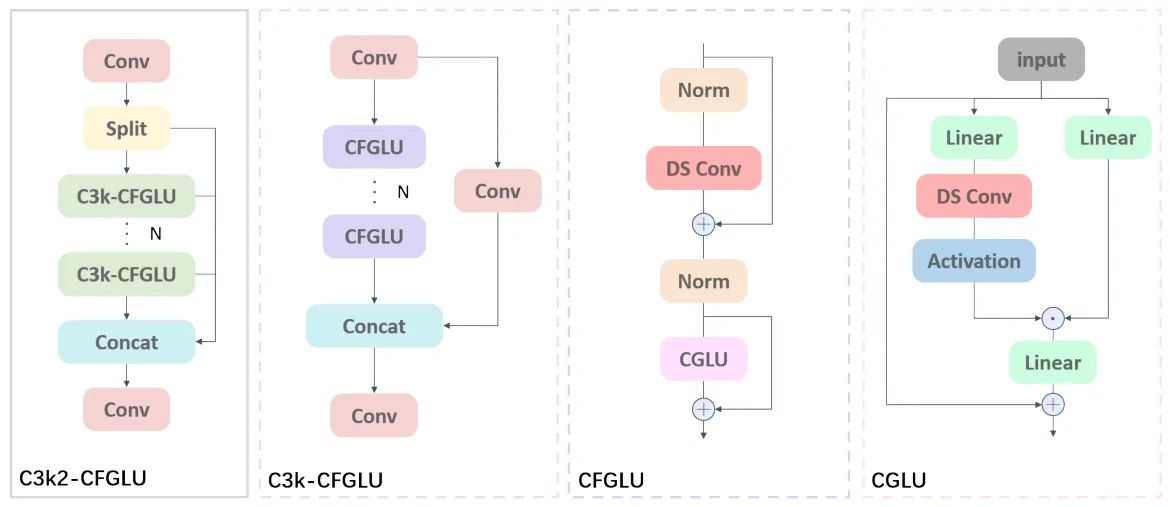
The network architecture of the C3k2-CFGLU model.

The C3k2-CFGLU module is a lightweight feature enhancement structure, as illustrated in Figure 5. Its core design concept is to enhance the representational power of convolutional neural networks by introducing advanced ideas from the vision Transformer paradigm, while strictly maintaining a low parameter count and efficient inference characteristics. The module is built upon the original multi-branch C3k2 architecture of YOLO11n, integrating two key components ConvFormer and CGLU [29] into its core feature extraction path. Among them, ConvFormer is inspired by the MetaFormer general architecture paradigm. By employing depthwise separable convolution as the Token Mixer, it simulates the spatial mixing capability of Transformers without introducing self-attention mechanisms, thereby effectively enhancing the perception accuracy of local details such as defect edges and textures on insulators. On this basis, the introduction of CGLU further strengthens the channel modeling capability of the model. By embedding depthwise convolution into the gating branch, the generation of gating signals at each spatial position is based on the local features of its neighborhood, enabling fine-grained dynamic modulation of channel weights. This achieves the effect of enhancing defect regions while suppressing background noise. Through the close integration of multi-branch parallel processing and feature fusion strategies, the C3k2-CFGLU module not only improves the recall rate and localization accuracy of small-scale defects but also reduces the model’s parameter count and computational cost, making it highly suitable for deployment on resource-constrained edge devices.

### 3.3. MBMSFPN Neck Network

In YOLO series detection models, the neck network undertakes the important task of fusing multi-scale features extracted by the backbone network and generating a more expressive feature pyramid. However, traditional path aggregation networks typically use simple addition or concatenation operations when fusing features of different scales, and the information paths are relatively fixed, which may result in insufficient and unbalanced utilization of shallow detail information and deep semantic information. To address this issue, the MBMSFPN structure was designed, aiming to construct a more efficient and flexible multi-scale feature fusion architecture.

In the bottom-up path, MBMSFPN not only fuses features from adjacent levels but also directly introduces shallower high-resolution information into deeper fusion nodes through additional skip connections. This aims to preserve and utilize the spatial details from the lowest levels of the backbone network, which is crucial for defect target detection. In the top-down path, feature fusion is also not limited to adjacent levels. A fusion node may simultaneously receive features from the same level, the level above, or even shallower levels. This multi-directional connection greatly enriches the semantic information and spatial details of the features. At each fusion node, features from different branches are not simply added together. MBMSFPN adopts the fast normalized fusion concept from BiFPN, introducing a learnable weight for each input feature. By normalizing these weights, the network can dynamically learn the importance of each input feature according to the training objective, achieving adaptive feature aggregation.

#### 3.3.1. MSCB Multi-Scale Convolution Block

**Figure 6:**
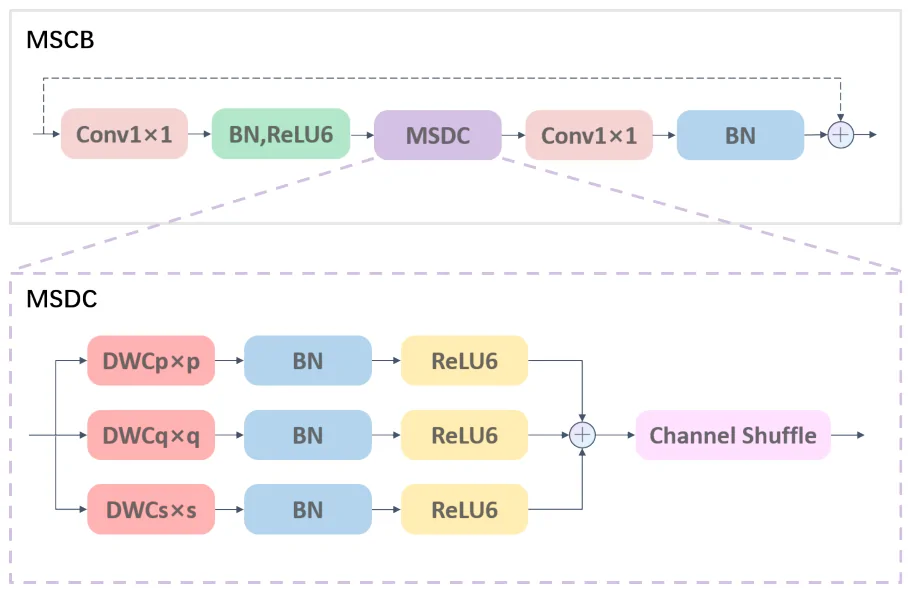
The network architecture of the MSCB model.

The Multi-Scale Convolution Block (MSCB) [30] plays a role in feature extraction and enhancement within MBMSFPN. Its structure is shown in Figure 6. The design innovates upon the inverted residual block (IRB) of MobileNetV2 by introducing multi-scale depthwise convolution (MSDC), which processes the same feature map in parallel using convolution kernels of different sizes, thereby capturing richer multi-scale contextual information. First, a 1 × 1 pointwise convolution expands the channel dimension of the feature map to increase feature capacity. The expanded feature map is then fed into the MSDC module, which internally consists of multiple depthwise convolution branches with different kernel sizes operating in parallel. Each branch focuses on extracting spatial features at a specific scale: small kernels focus on details, while large kernels focus on broader shapes. This design enables MSCB to adapt to the varying sizes of insulator defects, effectively responding to everything from small areas of damage or pollution-flashover to large areas of the insulator itself. The feature maps output by each branch are fused, and channel shuffling is performed to promote information exchange among different channel groups. Finally, another 1 × 1 convolution compresses the channel dimension back to the original size, outputting the enhanced feature map. MSCB makes extensive use of depthwise convolutions, whose parameter count and computational cost are much smaller than those of standard convolutions, which is key to maintaining its lightweight and efficient characteristics.

#### 3.3.2. EUCB Efficient Upsampling Block

**Figure 7:**
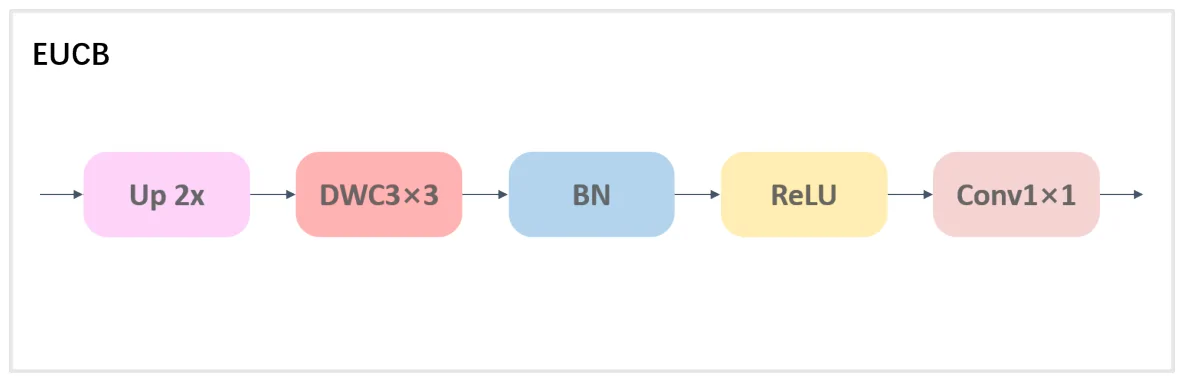
The network architecture of the EUCB model.

In the process of constructing a feature pyramid, low-resolution deep feature maps need to be upsampled to facilitate fusion with high-resolution shallow feature maps, giving rise to the upsampling module. However, traditional upsampling methods (such as transposed convolution) involve high computational cost and a large number of parameters, which are unfavorable for lightweight deployment. Therefore, this paper introduces the Efficient up-convolution block (EUCB) [30]. EUCB avoids computationally expensive transposed convolution and instead adopts a combination of lightweight upsampling and depthwise convolution, reducing computational overhead and parameter count while ensuring feature quality. Its structure is shown in Figure 7. First, a simple bilinear interpolation upsampling operation is applied to double the size of the feature map, achieving preliminary scale alignment. Subsequently, a 3 × 3 depthwise convolution processes the upsampled feature map to smooth the blocking artifacts caused by upsampling and enhance local detail information. Batch normalization and the ReLU activation function are employed to ensure training stability and nonlinear representation capability. Finally, a 1 × 1 convolution flexibly adjusts the channel dimension of the feature map to match the feature dimensionality required for the next stage.

### 3.4. SCDH Lightweight Detection Head

**Figure 8:**
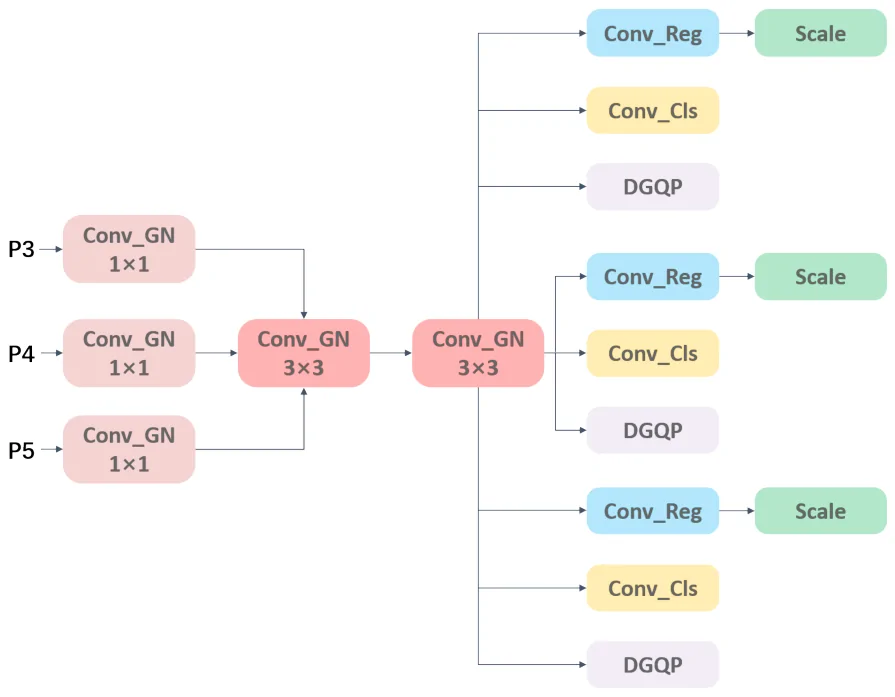
The network architecture of the SCDH Lightweight Detection Head.

In traditional detection heads, for the three scale feature maps output by the neck network, each scale branch possesses independent convolutional layer groups responsible for mapping input features to classification and regression outputs. SCDH breaks this tradition by allowing all scale branches to share the same set of convolution kernels, as shown in Figure 8. This means that regardless of the size of the feature map, the same convolutional weights are used for feature extraction. This design decouples the number of parameters in the detection head from the number of scales, considerably reducing model complexity. However, although shared convolutions are more efficient, feature maps at different scales naturally exhibit distributional differences. Directly using the same convolution kernels to process features at different scales may lead to training instability or performance degradation. To address this issue, this paper employs Group Normalization (GN) [31] to replace traditional Batch Normalization (BN). GN divides channels into several groups, computing the mean and variance independently within each group. Its performance is unaffected by batch size and is more stable in multi-scale scenarios with shared weights. In addition, a feature scaling layer is designed to adaptively adjust features after normalization, enabling the same set of convolution kernels to better accommodate the response intensities of feature maps at different scales, ensuring that both small and large targets obtain appropriate feature representations.

Finally, the Distribution-Guided Quality Predictor (DGQP) [32] is introduced to implement a localization quality estimation mechanism. This design models bounding box regression as a general distribution. Specifically, for the four sides of a bounding box (left, right, top, bottom), the network outputs a discrete probability distribution vector $P^{w}=\left[P^{w}\left(y_{0}\right),P^{w}\left(y_{1}\right),\ldots ,P^{w}\left(y_{n}\right)\right]$, where w∈{l,r,t,b}, indicating the probability that the position of the side falls within each discrete interval. The final regression value is given by the expectation of this distribution. The geometric interpretation of this distribution is that the shape and sharpness of the distribution reflect the uncertainty of the prediction. When the network is highly certain about the position of a side, the probability mass is highly concentrated on a few discrete values, forming a sharp distribution. Conversely, when the prediction is uncertain, the probability distribution is flatter. Experimental observations indicate a strong positive correlation between this statistical characteristic and the final localization quality: the sharper the distribution, the higher the localization quality; the flatter the distribution, the lower the localization quality.

The DGQP module first extracts statistical features. For the probability distribution vectors $P^{l},P^{r},P^{t},P^{b}$ of the four sides, DGQP extracts statistical features that reflect the sharpness of the distribution. In this paper, the Top-K maximum values and their mean are selected. When K = 4, the top four probability values and their mean are extracted for each side, yielding five statistics per side and a total of 20-dimensional feature vectors for the four sides.

(2) $$F=Concat\left(\left\{TopK\left(P^{w}\right)|w\in l,r,t,b\right\}\right).$$

where $TopK\left(P^{w}\right)$ denotes the joint operation of extracting the Top-K values and their mean. The advantage of this design is that the Top-K values directly reflect the concentration of the distribution; sharper distributions yield higher Top-K values. The mean provides auxiliary information and enhances representational capability. This feature is insensitive to absolute offsets in distribution positions and possesses good scale robustness.

The statistical feature vector F is then input into an extremely lightweight multi-layer perceptron (MLP):

(3) $$I=G\left(F\right)=\sigma \left(W_{2}\delta \left(W_{1}F\right)\right).$$

where δ is the ReLU activation function, σ is the sigmoid activation function, and $W_{1}$ and $W_{2}$ are learnable weight matrices. This MLP contains only one hidden layer, with a negligible number of parameters, resulting in minimal computational overhead for the overall model. The output I∈[0,1] is the predicted localization quality score.

Finally, the localization quality score I output by DGQP is element-wise multiplied with the class probability vector C output by the classification branch to obtain a joint representation:

(4) J=C×I.

This joint representation contains both class confidence and localization quality information. It is supervised by Quality Focal Loss (QFL) during training and is directly used as the sorting score for Non-Maximum Suppression (NMS) during inference. This decoupled fusion approach achieves higher accuracy and efficiency compared to directly concatenating distribution features and classification features to predict a joint score.

In summary, the SCDH module consists of a shared convolution structure synergistically combined with a localization quality estimation mechanism. Multi-scale shared convolutions and group normalization considerably reduce the number of parameters, while the localization quality estimation mechanism generates reliable localization quality scores through the lightweight DGQP using the statistical characteristics of bounding box distributions. This design ensures detection accuracy while considerably reducing computational overhead.

### 3.5. Training Objectives

To ensure the proposed FS-YOLO can be independently reproduced, we explicitly define the complete training objective. The overall loss function $L_{total}$ is a weighted sum of the Quality Focal Loss ($L_{QFL}$), the Distribution Focal Loss ($L_{DFL}$), and the GIoU Loss ($L_{GIoU}$). The composite loss is formulated as:

(5) $$L_{total}=\alpha \cdot L_{QFL}+\beta \cdot L_{DFL}+\gamma \cdot L_{GIoU},$$

where α, β, and γ are the loss weight coefficients, with values of 1, 0.25, and 2, respectively. In the SCDH module, the quality score *I* generated by DGQP is multiplied with the classification probability C to form the joint representation J=C×I. We adopt the Quality Focal Loss to supervise this continuous label J:

(6) $$L_{QFL}=-{\left|y-\sigma \right|}^{\beta }\cdot \left[y\log(\sigma )+(1-y)\log(1-\sigma )\right],$$

where σ is the predicted joint score, and y∈[0,1] is the target continuous label (IoU for positives, 0 for negatives). The focusing parameter β is set to 2.0.

For the four edges of the bounding box, the network predicts a discrete probability distribution $P(y_{i})$ over n+1 bins ($y_{0},y_{1},\ldots ,y_{n}$). Assuming the ground-truth target *y* lies between two adjacent bins $y_{i}$ and $y_{i+1}$, the Distribution Focal Loss is defined as:

(7) $$L_{DFL}\left(P,y\right)=-\left[\left(y_{i+1}-y\right)\log P\left(y_{i}\right)+\left(y-y_{i}\right)\log P\left(y_{i+1}\right)\right],$$

This loss encourages the network to concentrate probability mass on the bins adjacent to the true offset, thereby producing a sharp distribution that correlates with high localization quality.

The final regression value of the box is obtained by the expectation of the distribution. We minimize the GIoU loss between the predicted expectation and the ground-truth box to refine the localization accuracy:

(8) $$L_{GIoU}=1-\mathrm{GIoU}\left(b_{pred},b_{gt}\right).$$

where $b_{pred}$ and $b_{gt}$ denote the predicted and ground-truth bounding boxes, respectively.

### 3.6. LAMP Pruning Algorithm

In the field of neural network pruning, Magnitude Pruning (MP) is widely adopted due to its simplicity and efficiency. This method judges the importance of weights based on their absolute values, removing connections with smaller magnitudes. However, MP faces a key challenge: how to allocate reasonable compression ratio to different layers of the network. Global MP employs a single threshold for uniform pruning, which may cause some layers to be overly pruned or even completely zeroed out. Uniform MP applies the same compression ratio to all layers, ignoring the varying tolerance of different layers to parameter redundancy. Although existing heuristic methods can achieve certain results, they rely on manual experience and lack generality.

To overcome the above limitations, Lee et al. [33] proposed a pruning method named LAMP (Layer-Adaptive Magnitude-based Pruning). The core of LAMP is an importance score for global pruning, defined as follows: For a weight vector W in a given layer, sorted in ascending order of absolute values, the LAMP score of the u-th weight is:

(9) $$Score\left(u;W\right)=\frac{{\left(W\left[u\right]\right)}^{2}}{\sum_{v\geq u}{\left(W\left[v\right]\right)}^{2}},$$

The denominator represents the sum of squares of the current weight and all weights in the same layer with absolute values not less than that of the current weight. Therefore, the LAMP score measures the relative importance of this weight within the set of weights that have not yet been pruned in the current layer. Notably, the weight with the largest absolute value in each layer always has a LAMP score of 1, which ensures that at least one connection is retained per layer during global pruning, thereby avoiding the problem of entire layers being completely pruned away. The entire pruning process can dynamically adjust the pruning ratio by modifying the compression ratio $P_{r}$, expressed as:

(10) $$Pr=\frac{C_{pre}}{C_{pst}},$$

where $C_{pre}$ is the computational cost before pruning and $C_{pst}$ is the computational cost after pruning.

#### Structured LAMP Pruning Pipeline

Applying the LAMP score defined in Equation (9) to structured channel pruning requires a systematic pipeline that addresses three challenges: aggregating weight-level scores into channel-level importance, resolving channel dependencies in modern CNN architectures, and physically reconstructing the pruned model. For a convolutional layer with weight tensor $W\in R^{C_{\mathrm{out}}\times C_{\mathrm{in}}\times K\times K}$, the channel-level importance score $S_{k}$ for the *k*-th output channel is obtained by aggregating the LAMP scores of all weights in that channel:

(11) $$S_{k}=\sum_{i=1}^{C_{\mathrm{in}}}\sum_{u=1}^{K}\sum_{v=1}^{K}\mathrm{Score}\left(W\left[k,i,u,v\right]\right),$$

where Score(·) denotes the LAMP score defined in Equation (9). A global pruning threshold τ is then determined according to the target compression ratio $P_{r}$, and all channels with $S_{k}<\tau$ are marked for removal.

Channel dependencies arise from residual connections, concatenation operations, and depthwise convolutions in modern backbones such as YOLO11. For residual connections, the main path and shortcut path must be pruned with strictly aligned channel indices to preserve element-wise addition. For concatenation layers, pruning requires careful index remapping to ensure subsequent layers correctly reference the remaining channels. For depthwise convolutions, pruning masks must be applied consistently across input and output dimensions. To systematically handle these dependencies, we construct a dependency graph during model initialization, wherein each convolutional layer is a node and edges denote channel-wise coupling relationships. Once the pruning mask is determined for any layer, the graph propagates pruning decisions to all coupled channels, preserving architectural consistency.

Physical reconstruction of the pruned model proceeds as follows. For each convolutional layer, a new weight tensor $W^{\prime }\in R^{C_{\mathrm{out}}^{\prime }\times C_{\mathrm{in}}^{\prime }\times K\times K}$ is constructed by extracting only the rows and columns corresponding to unpruned channels. Concurrently, the associated Batch Normalization parameters—running mean, running variance, scale γ, and shift β—are extracted for the retained channels to form a reduced BN layer. The reconstructed layers are then reconnected according to the original topology with updated tensor shapes, ensuring direct executability without shape mismatches. Pruning is performed iteratively and the cycle repeats until the target $P_{r}$ is achieved.

### 3.7. Experimental Dataset

**Figure 9:**
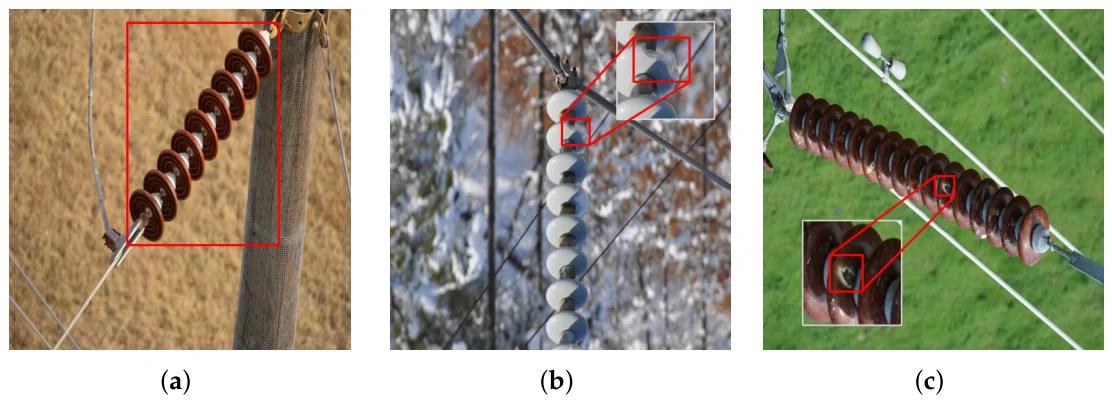
Representative samples of the dataset. (**a**) normal insulator; (**b**) damaged insulator; (**c**) pollution-flashover insulator.

The dataset used in this study integrates the high-quality public insulator dataset IDID, along with other publicly available insulator datasets (available at: https://universe.roboflow.com/uttam/insulator-defect/dataset/12) (accessed on 30 August 2026). After quality rechecking and annotation review, a total of 4192 images were obtained, of which 1600 were sourced from the IDID dataset. The dataset encompasses three categories: normal insulators, damaged insulators, and pollution-flashover insulators, with total instance counts of 7732, 3888, and 8180, respectively. Among these, the instance counts from the IDID dataset are 1093, 1832, and 2448, respectively. Example images are shown in Figure 9. All input images are resized to 640 × 640 pixels, with backgrounds covering various complex environments such as fields, mountains, skies, and icy conditions. A total of 4192 insulator defect images were randomly divided into training, validation, and test sets in a ratio of 7:2:1. Since publicly available insulator defect datasets are scarce and the number of high-quality samples collected in this study is limited, a series of data augmentation techniques were adopted during training to prevent overfitting caused by insufficient sample size. These techniques include vertical flipping, mirroring, brightness adjustment, translation, scaling, rotation, and Mosaic augmentation, all aimed at improving the model’s robustness and mitigating overfitting.

To ensure the validity of our evaluation, we conducted a perceptual hash (pHash) based near-duplicate analysis across the training, validation, and test splits. Following common practice, we adopted a Hamming distance threshold of 10 to identify near-duplicate images. The analysis revealed no cross-set duplicate pairs, confirming that our data splits are clean and the reported results are not inflated by data leakage.

**Figure 10:**
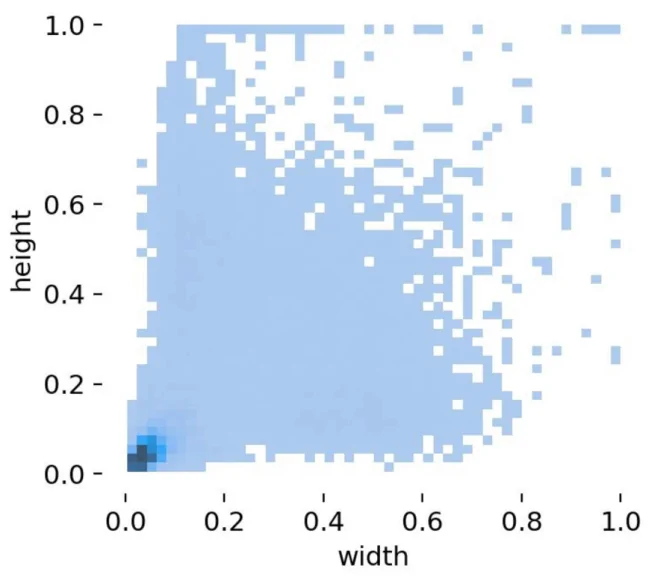
Two dimensional thermal distribution map of dataset samples.

Figure 10 illustrates the two-dimensional distribution of the normalized width and height of the defect instances. The overall distribution exhibits a pronounced left-skewed characteristic, with a substantial mass density predominantly concentrated near the origin (0.0, 0.0). Specifically, the majority of defects possess small spatial extents, characterized by width and height dimensions falling roughly within the range of 0.0 to 0.2. As these dimensions increase towards 1.0, the sample density declines drastically, resulting in sparse, scattered light-blue bins in the upper-right quadrant. Although larger anomalies are present in the dataset, they constitute a negligible proportion of the total samples. This highly skewed distribution, dominated by small-scale instances, underscores the inherent difficulty of the dataset and highlights the necessity of implementing specialized, robust algorithms tailored for defect object detection.

### 3.8. Experimental Environment

To ensure fairness and impartiality in the experiments, all models are trained for 200 epochs with a unified input image size of 640 × 640 pixels, a batch size of 16, and 4 data loading worker processes. The SGD optimizer is adopted with an initial learning rate of 0.01, a momentum factor of 0.937, and a weight decay coefficient of 0.0005. All experiments are conducted under three fixed random seeds (0, 1234, and 2025), and the reported results are averaged over these three independent runs. All models were evaluated using the standard evaluation procedure of the official Ultralytics YOLO framework, without any modification to the default evaluation settings. The detailed operating environment is provided in Table 1.

For inference speed measurements, all models used a batch size of 1 with a 200 iteration warm-up followed by 1000 iterations for timing, reporting the average latency per image under FP16 precision. CUDA synchronization was applied before each iteration, and the reported FPS includes preprocessing, inference, and NMS post-processing. This protocol was uniformly applied across all compared models.

To ensure the full reproducibility of our comparative experiments, we provide a detailed configuration summary for each baseline model in Table 2, including the exact repository source, software version, and model configuration file. All models were trained from scratch on our dataset without using pretrained weights.

### 3.9. Evaluation Metrics

To comprehensively and objectively evaluate the performance of the insulator defect detection model proposed in this paper, a commonly adopted evaluation metric system in the field of object detection is employed, assessing the model from two dimensions: detection accuracy and model complexity. Precision (P), Recall (R), mean Average Precision (mAP), and mAP@0.5:0.95 are adopted as the evaluation metrics in this paper. Their calculation formulas are as follows:

(12) $$P=\frac{TP}{TP+FP},R=\frac{TP}{TP+FN},$$

(13) $$AP=\int_{0}^{1}P\left(R\right)\;dR,$$

(14) $$mAP=\frac{1}{n}\sum_{i=1}^{n}AP_{i},$$

(15) $$mAP@0.5:0.95=\frac{1}{10}\sum_{i=1}^{10}\left(\frac{1}{n}\sum_{c=1}^{n}AP_{c,t_{i}}\right).$$

where TP denotes the number of correctly detected defect samples, FP denotes the number of samples where the background is incorrectly detected as defects, and FN denotes the number of missed defect samples. *n* represents the total number of detection categories. $AP_{c,t_{i}}$ represents the average precision for category *c* under the IoU threshold *t*.

This paper primarily adopts mAP@0.5 as the core evaluation metric, which is the mAP value calculated with an IoU threshold of 0.5. This metric reflects the model’s detection capability under lenient localization conditions. For the insulator defect detection task, defect sizes are generally small, demanding higher localization accuracy. Therefore, this paper also focuses on mAP@0.5:0.95, which is the average mAP value over IoU thresholds $t_{i}$ ranging from 0.5 to 0.95 with a step size of 0.05, to evaluate the model’s localization accuracy more strictly. In addition, the number of parameters and floating-point operations (FLOPs) are also adopted to reflect the model’s computational complexity.

## 4. Results and Discussion

### 4.1. Analysis of LAMP Experimental Results

In this study, an iterative channel pruning strategy is adopted instead of one-shot pruning. The pruning operations are performed on the weights of the fully trained and converged FS-YOLO base model, rather than on intermediate pre-training checkpoints. For each network layer, the pruning threshold is dynamically determined based on the distribution of the weight magnitudes in that layer. Meanwhile, the channel retention ratio is constrained on a per-layer basis to prevent excessive compression of the shallow layers, which play a critical role in extracting features of minor defects. After each round of channel pruning, the model is fine-tuned for 200 epochs using the identical insulator defect dataset employed in the initial training phase. The initial learning rate for post-pruning fine-tuning is set to 0.01, and the same learning rate decay schedule as in the original training is consistently applied throughout the fine-tuning process. This iterative pruning cycle is repeated until the preset overall model compression ratio is achieved. Under the condition that all hyperparameters are kept strictly identical across all rounds, a total of 16 comparative experiments are conducted with pruning rates $P_{r}$ ranging from 1.0 to 2.5, and the results are presented in Table 3.

Experimental results demonstrate that moderate pruning can improve the detection accuracy of the model. When the compression ratio $P_{r}=1.1$, the model achieves optimal accuracy, with mAP@0.5 reaching 93.2% and mAP@0.5:0.95 reaching 64.1%, representing improvements of 3.1% and 4.3%, respectively, over the matched unpruned control $\left(P_{r}=1.0\right)$. Meanwhile, precision and recall also increase to 93.1% and 88.7%, respectively. As the compression ratio further increases, the model accuracy shows a gradual declining trend. When $P_{r}\geq 1.5$, mAP@0.5 drops below 92.4%; when $P_{r}=2.0$, mAP@0.5 is 91.6%, a decrease of 1.6% from the optimal value; when $P_{r}=2.5$, mAP@0.5 further decreases to 89.5%, which is already lower than the matched unpruned control model level. These results indicate that excessive pruning impairs the model’s representational capacity, leading to considerable degradation in detection performance.

In terms of model complexity, as the compression ratio increases, the number of parameters, GFLOPs, and model file size all exhibit a monotonically decreasing trend. When $P_{r}=1.1$, the number of parameters decreases from 1.84 M to 1.34 M, a reduction of approximately 27.2%, and the model size is compressed from 4.0 MB to 3.0 MB, a reduction of 25.0%. When $P_{r}=1.5$, the number of parameters further decreases to 0.80M, a reduction of approximately 56.5%, and the model size is reduced to 2.0 MB, a reduction of 50.0%. When $P_{r}=2.5$, the model file size is only 1.3 MB, achieving a compression rate of 67.5%.

To achieve model compression and meet subsequent deployment requirements, this study selects a compression ratio of $P_{r}=1.4$ for model compression. It should be noted that this compression ratio was determined based on the detection performance on the validation set, which was held out from training. The test set was not involved in any stage of the pruning ratio selection and was used only for the final evaluation of the selected model. At this compression ratio, the number of model parameters is compressed from the original 1.84M to 0.88M, a reduction of approximately 52.2%; GFLOPs decrease from 5.7 to 4.1, a reduction of approximately 28.1%; and the model file size is reduced from 4.0 MB to 2.1 MB, achieving a compression rate of 47.5%. Meanwhile, the model still maintains high detection accuracy: mAP@0.5 is 92.9% and mAP@0.5:0.95 is 62.9%, reaching 99.7% and 98.1% of the optimal performance (achieved at $P_{r}=1.1$), respectively. The accuracy loss is controlled within an acceptable range while the model complexity is considerably reduced. In summary, $P_{r}=1.4$ achieves substantial model compression while ensuring detection performance, making it well-suited for deployment in resource-constrained scenarios.

**Figure 11:**
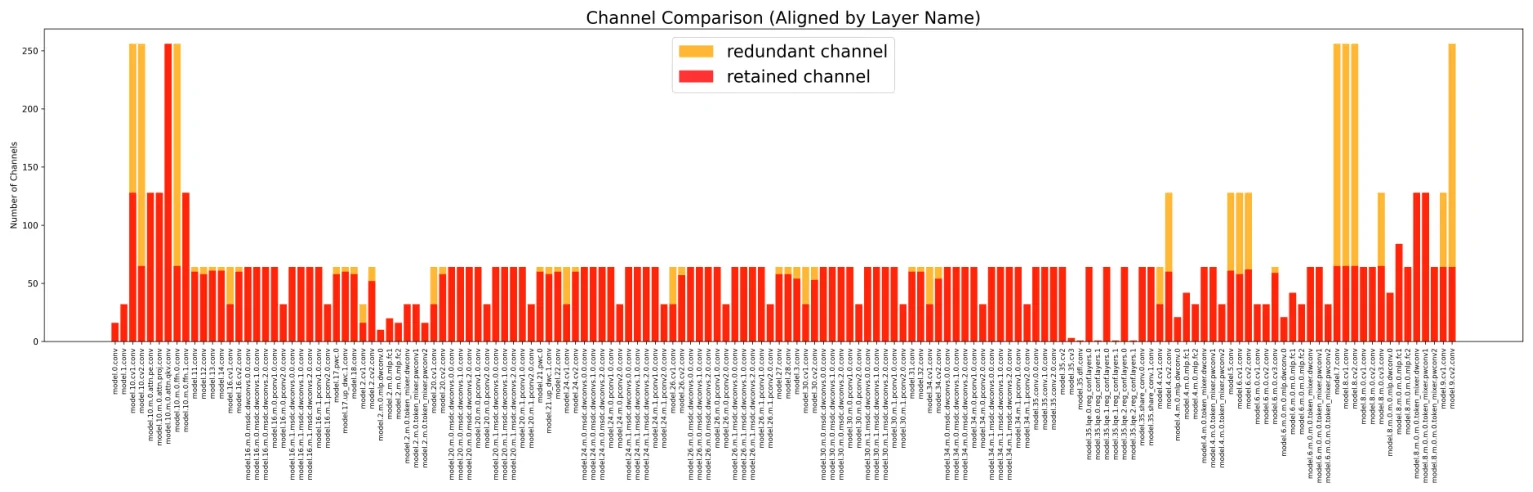
Comparison of channels before and after model pruning when Pr = 1.4.

To intuitively demonstrate the compression effect of the $P_{r}=1.4$ pruning strategy on the convolutional channels of each layer, this study presents the changes in channel counts before and after pruning, as shown in Figure 11. Among them, the orange channels represent the pruned redundant channels, while the red channels represent the retained channels.

### 4.2. Ablation Experiments

To validate the effectiveness of each proposed improvement module, ablation experiments are conducted on FS-YOLO. Using YOLO11n as the baseline model, the C3k2-CFGLU module, MBMSFPN module, SCDH module, and LAMP channel pruning are gradually introduced. The results are shown in Table 4. When the C3k2-CFGLU, MBMSFPN, and SCDH modules are added individually, mAP@0.5 improves by 0.8%, 0.4%, and 1.4%, respectively, compared with the baseline model, while mAP@0.5:0.95 improves by 0.9%, 1.0%, and 1.3%, respectively, indicating that each module considerably enhances the model’s ability to perceive insulator defects. When combinations of these three modules are tested, both mAP@0.5 and mAP@0.5:0.95 show improvements over the individual module configurations. In particular, when all three modules are added simultaneously, the model’s mAP@0.5 increases to 90.1%, while the parameter count and computational cost are reduced to 1.84 M and 5.7 GFLOPs, respectively. Compared with the baseline model, mAP@0.5 improves by 2.9%, mAP@0.5:0.95 improves by 2.6%, and the parameter count and FLOPs are reduced by 28.7% and 9.5%, respectively. Finally, LAMP channel pruning is introduced, further enhancing model performance while considerably reducing model complexity. mAP@0.5 and mAP@0.5:0.95 reach 92.9% and 62.9%, respectively, while the parameter count and computational cost are reduced to 0.88M and 4.1 GFLOPs, respectively. These results demonstrate that the improved model not only considerably enhances detection accuracy but also substantially reduces the parameter count and computational cost, providing an efficient and lightweight technical solution for intelligent edge-side inspection.

### 4.3. Comparative Experiments with Different Lightweight YOLO Models

In the comparative experiments, the general-purpose YOLO baselines were trained following the default training recipes of their official repositories, and the task-specific methods YOLOLS and LDDFSF-YOLO11 were trained with their published configurations as specified in the original papers. All models were evaluated under the identical evaluation protocol, and the results are shown in Table 5. In terms of precision and recall, FS-YOLO achieves a precision of 93.0% and a recall of 88.3%, both considerably outperforming the other compared models, indicating its more pronounced advantages in reducing missed detections and false alarms. In terms of the core detection accuracy metrics, FS-YOLO also performs the best, with mAP@0.5 reaching 92.9%, which is 5.7%, 6.2%, and 3.6% higher than YOLO11n, YOLO12n [34], and YOLO13n [35], respectively. The mAP@0.5:0.95 reaches 62.9%, which is 5.7%, 5.9%, and 3.7% higher than YOLO11n, YOLO12n, and YOLO13n, respectively. This metric requires a high degree of overlap between predicted boxes and ground-truth boxes under stricter IoU thresholds. FS-YOLO’s leading advantage demonstrates its higher precision in defect boundary localization. In terms of model complexity, FS-YOLO has only 0.88M parameters and 4.1 GFLOPs, making it the most lightweight among all compared models in terms of parameter count and model size. Compared with the baseline YOLO11n, the parameter count is reduced by 65.9% and the computational cost by 34.9%; compared with YOLO13n, the parameter count is reduced by 64.1% and the computational cost by 32.8%.

Furthermore, to substantiate the advantages of FS-YOLO over recent task-specific lightweight insulator defect detection methods, we additionally compared it with two representative methods in this field, namely YOLOLS and LDDFSF-YOLO11. Compared with YOLOLS, FS-YOLO improves precision by 0.7%, recall by 1.1%, mAP@0.5 by 0.2%, and mAP@0.5:0.95 by 1.8%, while reducing the parameter count by 30.7% and the model size by 25.0%, at a comparable computational cost. Compared with LDDFSF-YOLO11, FS-YOLO improves precision by 0.8%, recall by 0.7%, mAP@0.5 by 0.4%, and mAP@0.5:0.95 by 1.2%, while reducing the parameter count by 21.4%, the computational cost by 4.7%, and the model size by 19.2%. Meanwhile, FS-YOLO achieves an inference throughput of 171 FPS, which is 18.8% and 62.9% higher than YOLOLS and LDDFSF-YOLO11. From both detection accuracy and model complexity perspectives, FS-YOLO achieves the highest detection accuracy while having the smallest parameter count and model size, together with a computational cost (4.1 GFLOPs) comparable to that of the lightest compared model (YOLOLS, 3.9 GFLOPs). On the experimental platform used in this study (NVIDIA RTX A4000), its overall performance surpasses that of other mainstream lightweight YOLO models, demonstrating its favorable performance in insulator defect detection and indicating its potential for engineering deployment in resource-constrained scenarios.

**Figure 12:**
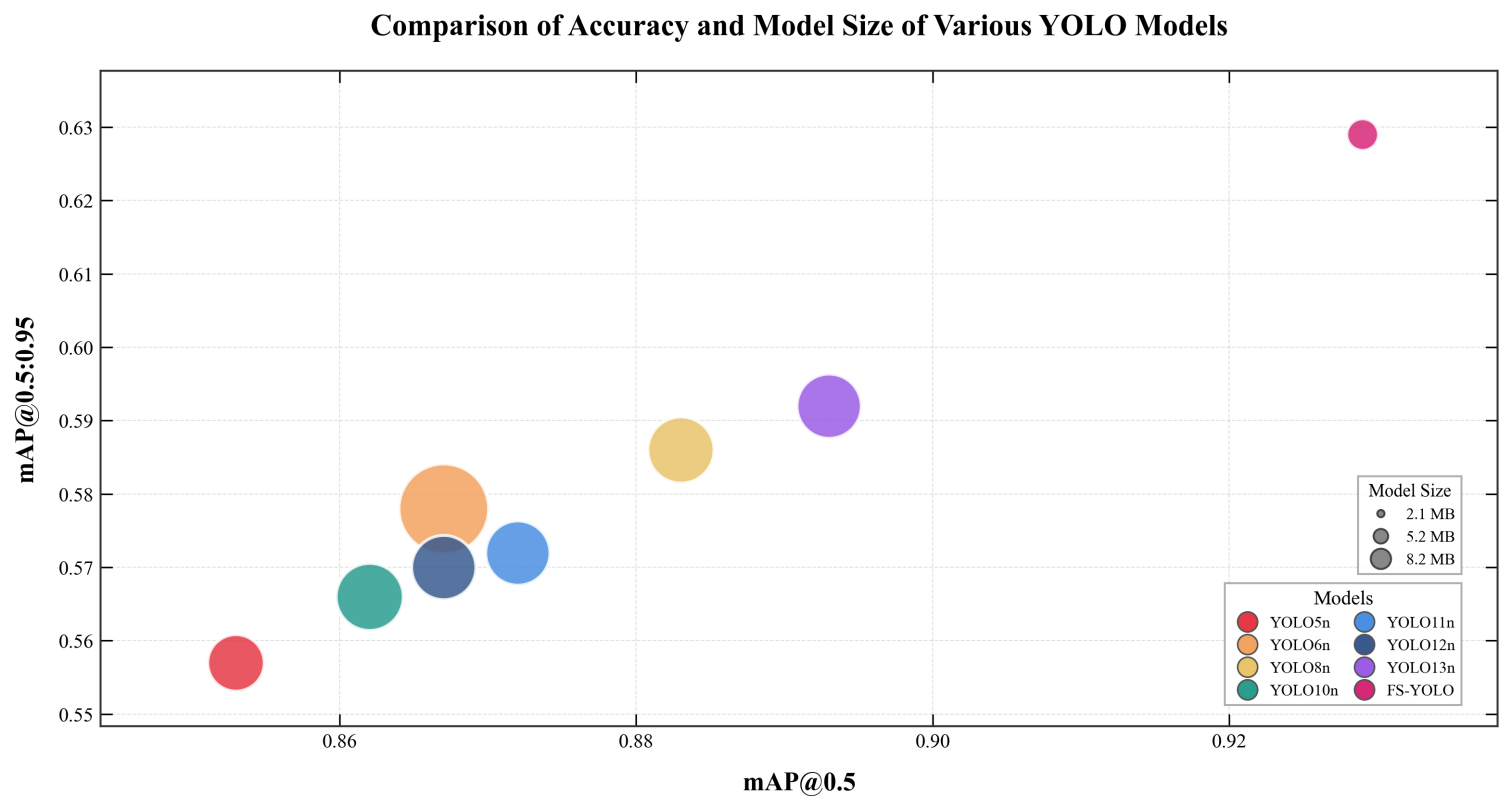
Comparison of FS-YOLO with various lightweight YOLO models in terms of accuracy and model size.

As shown in Figure 12, the overall performance of FS-YOLO compared with other mainstream lightweight YOLO models is presented more intuitively. The horizontal axis represents the mAP@0.5 metric, and the vertical axis represents the stricter mAP@0.5:0.95 metric. The size of each data point reflects the model size, demonstrating that FS-YOLO achieves a balance between deployment efficiency and detection accuracy.

**Figure 13:**
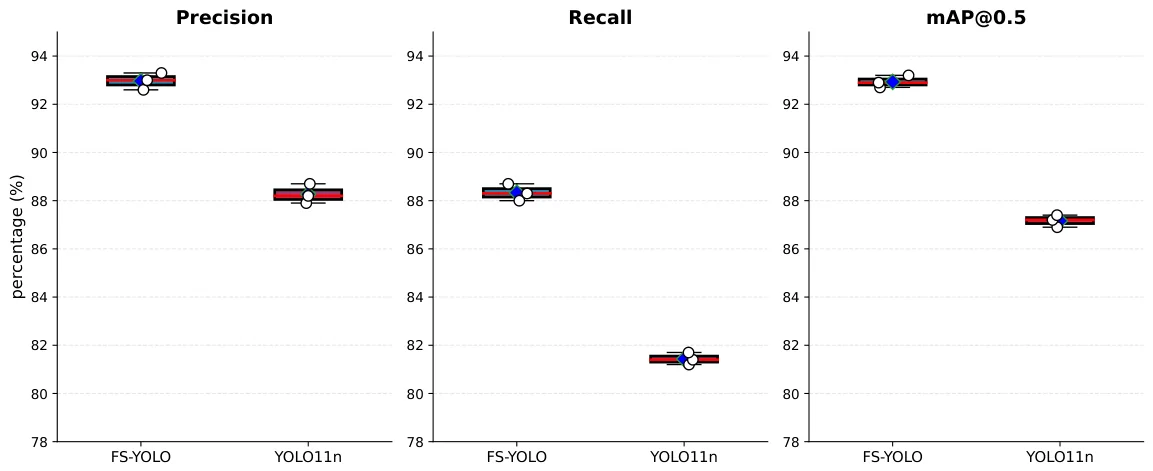
Boxplots comparing the performance of FS-YOLO and YOLO11n.

To evaluate the performance of FS-YOLO, we compared its statistical results with the baseline YOLO11n model using boxplots across Precision, Recall, and mAP@0.5. As illustrated in Figure 13, based on descriptive statistics, the absolute positions of the medians and means for FS-YOLO are consistently higher than those of YOLO11n across all three metrics. Furthermore, the boxes and whiskers of FS-YOLO are relatively narrower, indicating a smaller dispersion of sample data. Additionally, the ranges of data extremes between the two models do not overlap for any metric. It must be noted that this analysis is based purely on descriptive statistical observations without conducting inferential statistical tests; therefore, this figure cannot serve as inferential evidence proving statistically considerable differences in overall performance between the two models.

### 4.4. Comparative Analysis of Lightweight YOLO Models for Defect Targets

In actual transmission line inspection scenarios, insulator damage and pollution-flashover defects usually occupy a small number of pixels and contain weak visual features, imposing higher demands on the fine-grained representation capability of detection models. Since the dataset used in this study contains target objects such as normal insulators, to evaluate the advantages of FS-YOLO in defect target detection tasks, mainstream lightweight detection models are used as comparison baselines. The experimental results are shown in Table 6.

In the damage detection task, FS-YOLO demonstrates considerable advantages. It achieves a precision of 90.9%, a recall of 87.2%, mAP@0.5 of 91.8%, and mAP@0.5:0.95 of 55.2%, comprehensively outperforming all compared models across all four metrics. Compared with YOLO13n, the best-performing model among the compared methods, the proposed model improves precision by 2.1%, recall by 7.2%, mAP@0.5 by 4.5%, and mAP@0.5:0.95 by 4.4%. It is particularly noteworthy that the recall for damage detection reaches as high as 87.2%, indicating that FS-YOLO achieves an extremely low miss detection rate for this type of defect, which is of great significance for ensuring the safe operation of power systems.

In the pollution-flashover detection task, FS-YOLO also achieves optimal performance. It attains a precision of 92.2%, a recall of 80.6%, mAP@0.5 of 88.1%, and mAP@0.5:0.95 of 43.1%. Compared with YOLO13n, the best-performing model among the compared methods, FS-YOLO improves precision by 2.7%, recall by 7.9%, mAP@0.5 by 6.3%, and mAP@0.5:0.95 by 3.7%. The precision for pollution-flashover detection reaches as high as 92.2%, indicating that FS-YOLO achieves an extremely low false positive rate for this type of defect, effectively reducing the interference of false alarms on inspection operations.

### 4.5. Heatmap Visualization Comparative Experiment

**Figure 14:**
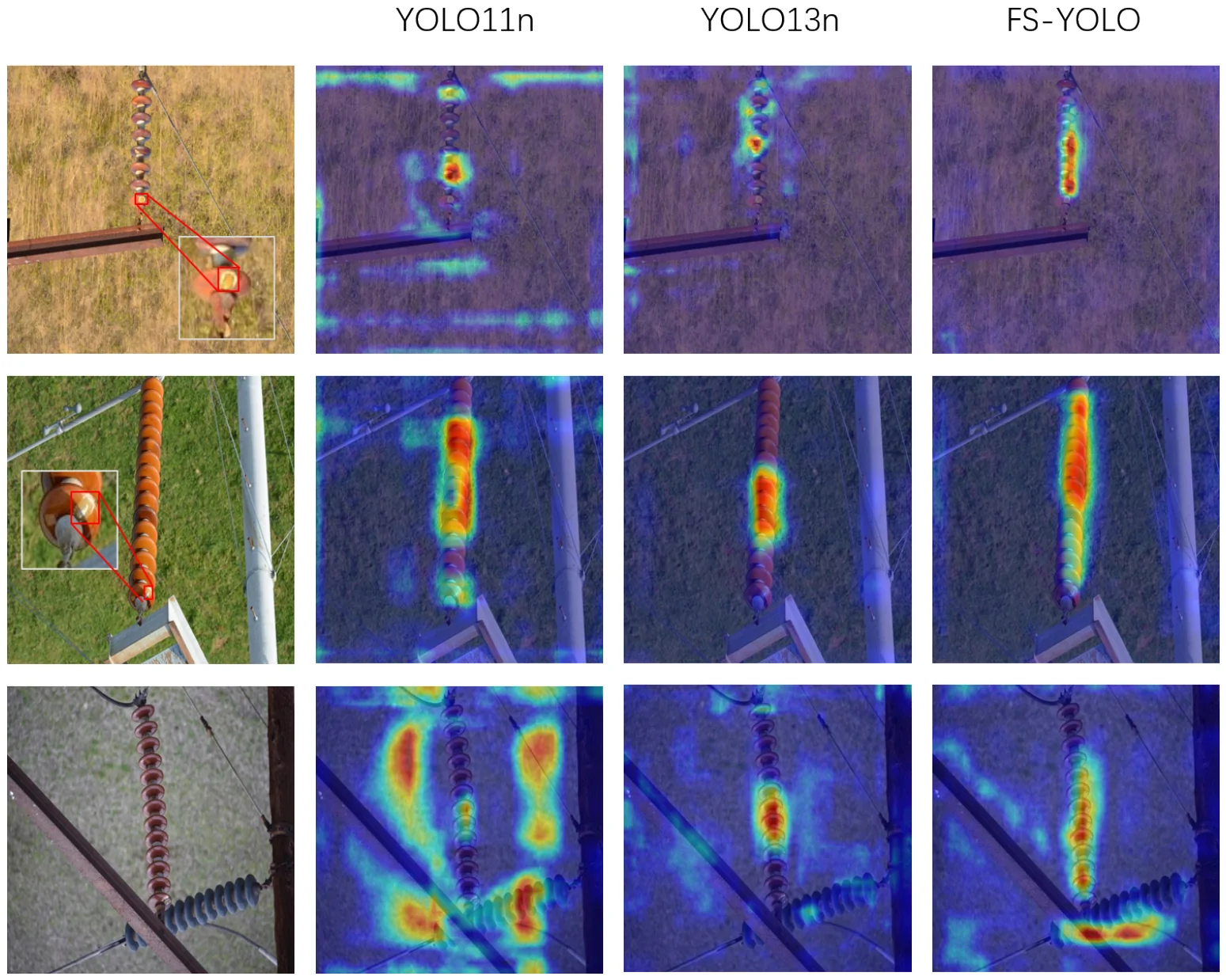
Comparison of heatmap effects among different models.

To further validate the effectiveness of the FS-YOLO model in target feature extraction, Figure 14 presents Grad-CAM [36] heatmaps of YOLO11n, YOLO13n, and FS-YOLO in various complex background and multi-scale target scenarios. In our experiments, all heatmaps were generated under strictly identical conditions to ensure a fair and unbiased comparison. The red regions in the heatmaps represent high-attention areas of the model in the current detection task, while the blue regions represent low-attention areas.

When there are abundant complex background textures and interfering objects, the heatmap distributions of YOLO11n and YOLO13n are relatively diffuse. High-activation regions appear not only on the target itself but also erroneously respond to background structures such as grass textures, metal crossarms, and towers. In contrast, FS-YOLO attention is highly concentrated on the insulator body, with almost no considerable activation response in the background regions. This indicates that FS-YOLO possesses stronger robustness to background interference, effectively reducing the false positive rate in complex backgrounds. When facing a single insulator body with relatively complete target integrity, although YOLO11n and YOLO13n localize the target, the activation region edges are blurred or even partially missing. In comparison, the activation region generated by FS-YOLO closely aligns with the contour of the insulator, with complete heatmap coverage, demonstrating that the model’s semantic perception of the overall target structure is more precise. Finally, in the task of detecting defects, both YOLO11n and YOLO13n exhibit attention shifts and fail to adequately cover the target. By contrast, FS-YOLO, while maintaining precise localization of large targets, can still effectively capture the features of such local defect targets, demonstrating the advantages of the model in multi-scale feature fusion.

The qualitative analysis of heatmaps shows that FS-YOLO can effectively focus on the key feature regions of targets, overcoming the issues of distracted attention in complex backgrounds, blurred target boundary responses, and missed detection of defect targets exhibited by YOLO series baseline models. This explains, from the perspective of feature learning, the considerable accuracy improvement of FS-YOLO observed in the above experimental results.

## 5. Conclusions

This paper proposes a lightweight insulator defect detection algorithm named FS-YOLO, based on multi-level feature enhancement and localization quality estimation, to address key issues in insulator defect detection tasks, such as large model parameter counts, insufficient defect target detection capability, and the vulnerability of existing models to interference in complex environments where insulators are located. The C3k2-CFGLU module enhances fine-grained feature representation while maintaining lightweight characteristics, improving the model’s robustness to background interference. The multi-scale and multi-branch design of MBMSFPN effectively improves the interaction efficiency between high-level and low-level features and enhances the perception of defect targets. SCDH compresses the parameter count through a shared convolution strategy and generates reliable localization quality scores using the statistical characteristics of bounding box distributions, thereby improving model detection performance. Finally, the LAMP channel pruning technique further compresses model complexity and enhances model performance.

In terms of experimental validation, the results from various experiments demonstrate that the proposed FS-YOLO achieves notable performance improvements. The precision and recall reach 93.0% and 88.3%, respectively, while mAP@0.5 and mAP@0.5:0.95 attain 92.9% and 62.9%, respectively, both improving by 5.7 percentage points over the baseline. Meanwhile, FS-YOLO maintains only 0.88M parameters and 4.1 GFLOPs, representing reductions of 65.9% and 34.9% compared with the baseline YOLO11n. These results validate the favorable balance between detection accuracy and computational efficiency achieved by FS-YOLO in the experimental environment (NVIDIA RTX A4000), indicating its potential for deployment in resource-constrained edge scenarios.

Future work will focus on the following directions. First, the insulator defect dataset used in this study is relatively limited, lacking samples of adverse weather conditions and more diverse types of anomalous insulators, which should be expanded to enhance the model’s practicality in more realistic scenarios. Second, the model will be migrated to other power equipment defect detection tasks to verify its generalization capability. Third, while the module-level ablation experiments in this study have sufficiently validated the effectiveness of each proposed module, a more granular subcomponent-level analysis could provide further insights into the architectural design. We plan to conduct such detailed ablation studies in our future work. Finally, the deployment and acceleration optimization of the model on devices such as UAVs and edge computing platforms in real-world scenarios will be explored, aiming to establish a more comprehensive and complete detection system.

## Figures and Tables

**Table 1:** Experimental platform environment.

Environmental Configuration	Version
GPU	NVIDIA RTX A4000
CPU	Intel core i9-12900k
RAM	64 GB
System	Windows 10
CUDA	12.1
Python	3.10
Torch	2.5.1

**Table 2:** Reproducibility configuration of compared baseline models.

Model	Repository Source	Version	Configuration File
YOLOv5n	https://github.com/ultralytics/yolov5 (accessed on 30 August 2026)	v7.0	yolov5n.yaml
YOLOv6n	https://github.com/meituan/YOLOv6 (accessed on 30 August 2026)	v3.0	yolov6n.yaml
YOLOv8n	https://github.com/ultralytics/ultralytics (accessed on 30 August 2026)	8.3.9	yolov8n.yaml
YOLO10n	https://github.com/ultralytics/ultralytics (accessed on 30 August 2026)	8.3.9	yolov10n.yaml
YOLO11n	https://github.com/ultralytics/ultralytics (accessed on 30 August 2026)	8.3.9	yolov11n.yaml
YOLO12n	https://github.com/ultralytics/ultralytics (accessed on 30 August 2026)	8.3.9	yolov12n.yaml
YOLO13n	https://github.com/ultralytics/ultralytics (accessed on 30 August 2026)	8.3.9	yolov13n.yaml

**Table 3:** Experimental results of FS-YOLO under different pruning ratios.

*Pr*	Precision	Recall	mAP@0.5	mAP@0.5:0.95	Params	FLOPs	Size
	(%)	(%)	(%)	(%)	(M)	(G)	(MB)
1.0	91.8	83.7	90.1	59.8	1.84	5.7	4.0
1.1	93.1	88.7	93.2	64.1	1.34	5.2	3.0
1.2	93.4	87.8	93.1	63.5	1.07	4.7	2.5
1.3	93.4	88.2	92.9	63.3	0.95	4.4	2.3
1.4	93.0	88.3	92.9	62.9	0.88	4.1	2.1
1.5	92.4	87.6	92.4	62.7	0.80	3.8	2.0
1.6	92.2	87.8	92.2	62.1	0.74	3.5	1.9
1.7	92.2	87.9	92.3	61.9	0.68	3.3	1.8
1.8	92.8	87.0	92.0	61.4	0.65	3.2	1.7
1.9	92.6	85.9	91.7	61.4	0.60	2.9	1.6
2.0	92.0	85.8	91.6	61.0	0.57	2.8	1.6
2.1	91.5	85.3	91.1	60.4	0.54	2.7	1.5
2.2	91.3	84.9	91.0	60.2	0.51	2.5	1.4
2.3	90.0	86.0	90.8	59.8	0.49	2.4	1.4
2.4	90.6	84.4	90.0	59.4	0.47	2.3	1.4
2.5	89.8	84.0	89.5	58.7	0.45	2.2	1.3

**Table 4:** Ablation experiment results.

C3k2-CFGLU	MBMSFPN	SCDH	LAMP	mAP@0.5	mAP@0.5:0.95	Params	FLOPs
				(%)	(%)	(M)	(G)
-	-	-	-	87.2 ± 0.21	57.2 ± 0.18	2.58	6.3
✓	-	-	-	88.0 ± 0.19	58.1 ± 0.22	2.31	6.1
-	✓	-	-	87.6 ± 0.25	58.2 ± 0.20	2.12	6.5
-	-	✓	-	88.6 ± 0.17	58.5 ± 0.15	2.42	5.6
✓	✓	-	-	88.6 ± 0.23	58.5 ± 0.19	1.98	6.4
✓	-	✓	-	89.8 ± 0.15	59.4 ± 0.21	2.16	5.4
-	✓	✓	-	88.9 ± 0.22	58.8 ± 0.17	1.98	5.9
✓	✓	✓	-	90.1 ± 0.23	59.8 ± 0.18	1.84	5.7
✓	✓	✓	✓	**92.9 ± 0.16**	**62.9 ± 0.14**	**0.88**	**4.1**

Note: ✓ signifies that the module is included, whereas - denotes that the module is excluded. Values in bold signify the optimal results.

**Table 5:** Comparative experiment of various lightweight YOLO models.

Model	Precision	Recall	mAP@0.5	mAP@0.5:0.95	Params	FLOPs	Size	FPS
	(%)	(%)	(%)	(%)	(M)	(G)	(MB)	
YOLOv5n	86.8 ± 0.22	79.4 ± 0.25	85.3 ± 0.28	55.7 ± 0.23	2.18	5.8	4.4	255
YOLOv6n	90.4 ± 0.25	80.3 ± 0.28	86.7 ± 0.22	57.8 ± 0.30	4.16	11.5	8.2	266
YOLOv8n	90.7 ± 0.18	82.9 ± 0.29	88.3 ± 0.20	58.6 ± 0.25	2.69	6.8	5.4	268
YOLO10n	86.6 ± 0.25	81.2 ± 0.32	86.2 ± 0.31	56.6 ± 0.24	2.7	8.2	5.5	**277**
YOLO11n	88.2 ± 0.22	81.4 ± 0.26	87.2 ± 0.21	57.2 ± 0.18	2.58	6.3	5.2	214
YOLO12n	89.2 ± 0.27	80.3 ± 0.24	86.7 ± 0.26	57.0 ± 0.23	2.51	5.8	5.2	187
YOLO13n	91.5 ± 0.20	82.3 ± 0.22	89.3 ± 0.18	59.2 ± 0.22	2.45	6.1	5.2	161
YOLOLS [18]	92.3 ± 0.14	87.2 ± 0.16	92.7 ± 0.14	61.1 ± 0.12	1.27	**3.9**	2.8	144
LDDFSF-YOLO11 [22]	92.2 ± 0.13	87.6 ± 0.19	92.5 ± 0.17	61.7 ± 0.16	1.12	4.3	2.6	105
FS-YOLO	**93.0 ± 0.17**	**88.3 ± 0.21**	**92.9 ± 0.16**	**62.9 ± 0.14**	**0.88**	4.1	**2.1**	171

Note: Values in bold signify the optimal results. FPS denotes the inference throughput measured with a batch size of 1, the corresponding per-image latency is approximately 1000/FPS ms.

**Table 6:** Comparison of various lightweight YOLO models on defect target detection.

Model	Precision (%)		Recall (%)		mAP@0.5 (%)		mAP@0.5:0.95 (%)		Params (M)	FLOPs (G)
	Damage	Flashover	Damage	Flashover	Damage	Flashover	Damage	Flashover		
YOLOv5n	78.5	86.3	72	69.9	79.1	78.3	42.6	36.3	2.18	5.8
YOLOv6n	84.7	89.8	75.5	69.1	81.5	80	47	37.6	4.16	11.5
YOLOv8n	88.4	87.3	79.1	72.9	86	80	48.5	38.2	2.69	6.8
YOLO10n	83	83.8	75	72.2	81.6	78.9	45.4	36.5	2.7	8.2
YOLO11n	81.9	87.3	75.8	71.4	82.6	80	45.5	37.2	2.58	6.3
YOLO12n	84.9	86.8	75.3	68.7	83.5	77.7	46.1	36.4	2.51	5.8
YOLO13n	88.8	89.5	80	72.7	87.3	81.8	50.8	39.4	2.45	6.1
FS-YOLO	**90.9**	**92.2**	**87.2**	**80.6**	**91.8**	**88.1**	**55.2**	**43.1**	**0.88**	**4.1**

Note: The best results are highlighted in bold and adopt abbreviated forms for the two defect categories, namely ‘damage’ and ‘flashover’.

## Data Availability

GitHub repository link: https://github.com/Wen-CJ/FS-YOLO (accessed on 16 August 2026). The implementation is based on PyTorch 2.5.1 and ultralytics 8.3.9.
